# Does lavender aromatherapy alleviate premenstrual emotional symptoms?: a randomized crossover trial

**DOI:** 10.1186/1751-0759-7-12

**Published:** 2013-05-31

**Authors:** Tamaki Matsumoto, Hiroyuki Asakura, Tatsuya Hayashi

**Affiliations:** 1 Department of Health Education, Faculty of Education, Shitennoji University, 3-2-1 Gakuenmae, Habikino Osaka, 583-8501, Japan; 2 Ohgimachi Ladies Clinic, Center for Advanced Reproductive Endocrinology and Infertility, Osaka, Japan; 3 Graduate School of Human and Environmental Studies, Kyoto University, Kyoto, Japan

## Abstract

**Background:**

A majority of reproductive-age women experience a constellation of various symptoms in the premenstrual phase, commonly known as premenstrual syndrome (PMS). Despite its prevalence, however, no single treatment is universally recognized as effective, and many women turn to alternative approaches, including aromatherapy, a holistic mind and body treatment. The present study investigated the soothing effects of aromatherapy on premenstrual symptoms using lavender (*Lavandula angustifolia*), a relaxing essential oil, from the perspective of autonomic nervous system function.

**Methods:**

Seventeen women (20.6 ± 0.2 years) with mild to moderate subjective premenstrual symptoms participated in a randomized crossover study. Subjects were examined on two separate occasions (aroma and control trials) in the late-luteal phases. Two kinds of aromatic stimulation (lavender and water as a control) were used. This experiment measured heart rate variability (HRV) reflecting autonomic nerve activity and the Profile of Mood States (POMS) as a psychological index before and after the aromatic stimulation.

**Results:**

Only a 10-min inhalation of the lavender scent significantly increased the high frequency (HF) power reflecting parasympathetic nervous system activity in comparison with water (aroma effect: *F* = 4.50, *p* = 0.050; time effect: *F* = 5.59, *p* = 0.017; aroma x time effect: *F* = 3.17, *p* = 0.047). The rate of increase in HF power was greater at 10–15 min (*p* = 0.051) and 20–25 min (*p* = 0.023) in the lavender trial than in the control trial with water. In addition, POMS tests revealed that inhalation of the aromatic lavender oil significantly decreased two POMS subscales—depression–dejection (*p* = 0.045) and confusion (*p* = 0.049)—common premenstrual symptoms, in the late-luteal phase, as long as 35 min after the aroma stimulation.

**Conclusions:**

The present study indicated that lavender aromatherapy as a potential therapeutic modality could alleviate premenstrual emotional symptoms, which, at least in part, is attributable to the improvement of parasympathetic nervous system activity. This study further implies that HRV could evaluate the efficacy of aromatherapy using various fragrances to relieve premenstrual symptoms, and ultimately, support the mind and body health of women.

## Background

A majority of women experience at least some degree of a regular recurrence of various biopsychosocial symptoms during the days prior to menstruation, which usually abate following menstruation. The cluster of symptoms can alter behavior and well-being, and affect family, friends, and working relationships. We commonly know this enigmatic condition appears in the late-luteal phase as premenstrual syndrome (PMS) [1-3]. Problematic symptoms of PMS may begin in the adolescent years, although the most severe symptoms occur in the 20s to mid-30s [1]. Symptoms and discomfort levels of PMS vary from woman to woman. Researchers have attempted to unveil this unique condition from various perspectives. At the time of writing, however, no consensus had emerged on which specific physical findings, biological markers, or laboratory tests comprise a diagnosis and/or etiopathogenesis of PMS [1-3]. Apart from complete elimination of the menstrual cycle, no single treatment is universally recognized as effective, and many women often turn to therapeutic approaches outside of conventional medicine [4].

The practice of aromatherapy—a form of complementary and alternative medicine—uses volatile plant materials, known as essential oils, and, with its ancient roots, has a long history of use in supporting women’s health and lifestyles [5,6]. While some predominantly anecdotal therapeutic evidence derives from small trials and case studies, aromatherapy has gained a resurgence of interest as women seek safe and efficacious options for their healthcare. Lavender, a representative of relaxing essential oils, enjoys popularity and wide use in the clinical fields of psychosomatic obstetrics and gynecology. Therapeutic treatments with lavender oil help women cope with labor pain [6], ease post-cesarean pain [7], reduce postpartum depression and anxiety [8], decrease dysmenorrhea [9], and alleviate climacteric symptoms including melancholia, hot flushes, arthralgia, and myalgia [10]. A short term of inhalation of essential oils containing linalool and linalyl acetate—two major components of lavender—also improves mood states during pregnancy [11] and insomnia among midlife women [12]. Taking these findings into consideration, we find it plausible that aromatherapy with lavender fragrance could ameliorate PMS. A literature search for this study using the PubMed database, a service of the US National Library of Medicine and the National Institutes of Health, however, identified no available empirical research regarding the efficacy of lavender on PMS.

Although all body systems contribute, the relative stability of the human internal environment depends largely on the orchestrations of the autonomic nervous system, comprising two divisions—sympathetic and parasympathetic nervous systems. Instability, or even a slight disorder of the autonomic nervous system, therefore, could induce broadly ranging psychophysiological phenomena. Bio-fluctuomatics technology allows us to explore the functioning of the autonomic nervous system reliably and noninvasively using comprehensive and functional analysis of heart rate variability (HRV). Researchers have consistently observed reduced HRV during and following acute and chronic stress [13,14]. Men and women exhibiting depression [15], anxiety [16], post-traumatic stress disorder [17], and chronic fatigue syndrome [18] also experience diminished HRV. Thayer et al. [19], in addition, found emotion regulation linked to HRV, i.e., individuals with greater emotion-regulation ability have been shown to have greater levels of resting HRV. Studies have investigated psycho-physiological effects of aromatherapy by using HRV and demonstrated significant changes of autonomic nervous system activity after inhalation of essential oils, including lavender, while affecting mood, mental states and behavior [20]. These findings demonstrate the functionality of HRV measurements to evaluate effects of aromatherapy on premenstrual symptomatology from the perspective of autonomic nervous system activity as it reflects mind and body interaction.

Accordingly, the present study designed a randomized crossover study to evaluate the efficacy of commercially available lavender oil on mood states and autonomic nervous system activity by using HRV measurements in the late-luteal phase of eumenorrheic women. We then investigated whether inhalation of lavender fragrance could serve as an alternative method to alleviate undefined psychoemotional conditions most reproductive-age women experience premenstrually.

## Methods

### Subjects

Seventeen women in their 20s volunteered to participate in this study. The women, all college students, responded to a campus advertisement. The study protocol was approved in advance by the Institutional Review Board of Shitennoji University and was performed in accordance with the Declaration of Helsinki of the World Medical Association. All subjects received an explanation of the nature and purpose of the study: to investigate soothing effects of plant fragrance on emotional symptoms in the premenstrual phase. We did not, however, inform subjects of which fragrance we would use for the experiments. Prior to receiving any data about the experiments, all subjects gave their written informed consent to participate in the study.

The subjects underwent medical examinations and interviews and completed a standardized health questionnaire regarding medical history, medications, current health condition, regularity of menstrual cycle, premenstrual discomfort, and lifestyle. While referring to subjects’ self-reported regular menstrual cycles, we determined the cycle phase during the experiments by the onset of menstruation and oral temperature verified by concentrations of ovarian hormones, estrone (E1), and pregnanediol-3-glucuronide (PdG), in a urine sample taken early in the morning. Both E1 and PdG were indexed to creatinine (Cr) excretion [21-24].

As to premenstrual discomfort, responses of participants to the health questionnaire indicated that all women had some subjective psychophysiological complaints of varying degrees, from mild to moderate. However, none of the women reported that premenstrual symptoms markedly interfered with work, school, usual activities or relationships with others. To determine the severity of premenstrual symptoms, we asked subjects to record a daily symptom diary based on the menstrual distress questionnaire [25] for at least two menstrual cycles. The average value of the increase on the total scores of the diary from the follicular (day 5 to day 11 from the first day of menstruation) to the late-luteal phase (within seven days before the next menstruation) was 10.3 ± 2.4%. None of the subjects reported greater than a 30% increase, a standard for diagnosing PMS [3,26]. Notably, according to a series of studies by the authors of this paper [21-24], a less than 20% increase of subjective symptoms from the follicular to the late-luteal phase did not influence autonomic nervous system activity during the menstrual cycle. Considering this information, no subjects in the present study suffered from severe PMS or premenstrual dysphoric disorder (PMDD), but the subjects’ symptoms did fall within the sphere of premenstrual molimina (subclinical levels of premenstrual symptomatology), signaling impending normal menstruation, which a majority of reproductive-age women experience [3,26].

None of the subjects had been clinically diagnosed with diabetes mellitus, hypertension, hyperlipidemia, or cardiovascular or any other endocrine or systemic disorders that could affect the autonomic nervous system. The subjects were non-obese and non-smokers. None of the women reported taking oral contraceptives to control the menstrual cycle. We did not perform a pregnancy test for this study; however, regular menstrual cycles subsequently resumed in all subjects after the completion of the study. Thus, results showed that none of the subjects were pregnant during the study period.

Referring to a study of Kiecolt-Glaser et al. [27], we performed the olfactory function test on all subjects to assure that none had anosmia. Briefly, subjects were given two sets of three bottles—two held distilled water; the third contained essential oils (lavender or orange)—and were asked to choose the one that differed from the other two. To be eligible for the study, subjects had to choose the correct response in both trials.

All subjects were asked not to consume any food or beverages containing alcohol or caffeine after 21:00 of the day preceding the experiment. The subjects were also instructed to abstain from alcohol use and excessive physical activity for 24 hours before testing [21].

### Experimental procedure

All subjects were examined on two separate occasions (aroma and control trials) in the late-luteal phase (within seven days before the next menstruation). The order of testing was randomized so that equal numbers of subjects were studied first in each trial. All measurements were taken between 11:00 and 15:00 and were performed in a temperature-controlled (25°C), quiet, comfortable room with a minimization of arousal stimuli. Height and body weight of each subject were measured to calculate body mass index (BMI) as body weight divided by height squared. Subjects then rested for at least 10 minutes before the start of the experiment.

This experiment used two kinds of aroma stimulation: lavender (*Lavandula angustifolia*, Lot No. BLAH10, Kensoigakusha Co. Tokyo, Japan) and water as a control. Major components of the lavender oil used in this study comprised linalyl acetate (37.18%), linalool (36.83%), trans-β-ocimene (4.25%), β-caryophyllene (3.55%), cis-β-ocimene (2.88%), and lavendulyl acetate (2.06%). Referring to previous studies [28,29], we pipetted 10 μl of lavender essential oil or water onto a small cotton pad designed for a diffuser (Aroma breeze NOVA T, ALTA Corporation, Nagoya, Japan). Airflow from the diffuser was set at 1.3 m per min and placed near the subject’s nostrils using the diffuser’s 30 cm long circular cylinder fitted with a perforated funnel (diameter 5 cm).

Before measurements were taken, the subjects were instructed to relax quietly and comfortably for at least 10 min in a seated position while equipped with electrocardiograph (ECG) electrodes. They then filled out the Profile of Mood States (POMS) explained in detail below. The ECG was recorded 5 min before inhalation of the scent. Each subject inhaled the scent for 10 min. We then measured the ECG for 5 min at 0, 10, 20, and 30 min after inhalation. During ECG recording, all subjects breathed in synchrony to a metronome at 15 beats per minute to ensure that the respiratory-linked variations in heart rate did not overlap with low-frequency heart-rate fluctuations (below 0.15 Hz) from other sources [21,23]. After the ECG was recorded, the subjects repeated the POMS test. The ECG signals were later analyzed by means of HRV power spectral analysis, as described below, to evaluate whether aroma stimulation changed autonomic nervous system activity.

### R-R interval power spectral analysis procedure

The autonomic nervous system activity was noninvasively measured by HRV power spectral analysis, which decomposes the series of sequential R-R intervals into a sum of sinusoidal functions of different amplitudes and frequencies by the Fourier transform algorithm. The technique of the analysis for the present investigation has been applied in basic physiological and clinical research fields, and its validity and reliability has been previously confirmed [21,23,30-32]. Researchers have elsewhere described the procedure of R-R interval power spectral analysis used in the present study in great detail [30,31]. Briefly, the ECG signal was amplified (MEG-6108, Nihon Kohden, Tokyo, Japan) and digitized via a 16-bit analog-to-digital converter (Model PS-2032GP, TEAC, Tokyo, Japan) at a sampling rate of 1000 Hz. The digitized ECG signal was differentiated, and the resultant QRS spikes and the intervals of the impulses (R-R intervals) were stored sequentially on a hard disk for later analyses.

Before the R-R spectral analysis was performed, the stored R-R interval data were displayed and aligned sequentially to obtain equally spaced samples with an effective sampling frequency of 2 Hz and displayed on a computer screen for visual inspection. Then, the direct current component and linear trend were completely eliminated by digital filtering for the band-pass between 0.03 and 0.5 Hz. The root mean square value of the R-R interval was calculated as representing the average amplitude. After passing through the Hamming window, power spectral analysis by means of a fast Fourier transform was performed on a consecutive 256-sec time series of R-R interval data obtained during the test. Spectral powers were calculated for the following respective frequency band: low frequency (LF) power (0.03–0.15 Hz), an indicator of both sympathetic and parasympathetic nervous system activity; high frequency (HF) power (0.15–0.5 Hz), which solely reflects parasympathetic nerve activity; and Total power (0.03–0.5 Hz) representing overall autonomic nervous system activity.

Basal heart rates and autonomic nervous system activities differ from individual to individual. Thus, the mean values for heart rates before inhalation of scent were set as the baseline values and the mean values for autonomic nervous system activity before the inhalation were standardized to 100%. The rate of change after the inhalation was compared between aroma and control trials [28].

### Assessment of emotional symptoms

We administered the Japanese version of the POMS test (Kaneko Shobo Co., Tokyo, Japan), a globally standardized, self-administered, 65-item questionnaire (including 7 dummy items) to assess premenstrual mood states before and after inhalation of the lavender aroma and water. Each item was rated on a five-point Likert-type scale of zero to four, ranging from “not at all” to “extremely.” We added these raw scores to generate six subscales of emotional state: tension–anxiety, depression–dejection, anger–hostility, vigor, fatigue, and confusion. These added raw scores were then converted into T-scores according to the POMS manual [33]. We should mention that, according to our recent study [24], five negative POMS variables—tension–anxiety, depression–dejection, anger–hostility, fatigue, and confusion—reflect the cluster of premenstrual psychoemotional symptoms. In addition, those variables significantly and positively correlated in the late-luteal phase. Referring to a study by Kuroda et al. [28], to investigate the effect of lavender aroma on mood states, we compared changes in the POMS scores of the lavender and control trials before and after the ECG measurements.

### Statistical analysis

To investigate the acute influence of inhalation of the lavender aroma on HRV spectral power, the effects of aroma and time and their interaction (aroma x time) were evaluated using two-way ANOVA with repeated measures. When significant interactions were found, we conducted paired t tests between lavender and control trials and one-way ANOVA with repeated measures during each trial. When Mauchly’s test of sphericity showed significance, probability values were adjusted using the Huynh-Feldt correction. Paired t tests were performed to compare changes in scores of the POMS test before and after the ECG measurements between aroma and control trials. Values are reported as mean ± standard errors (SE). *P* values < 0.05 were considered statistically significant. All statistical analysis was performed using a commercial software package (IBM SPSS Statistics Version 20).

## Results

### Clinical characteristics of subjects

Mean values of physical features of all subjects were as follows: age 20.6 ± 0.2 years, height 156.4 ± 1.5 cm, weight 52.5 ± 1.6 kg, and BMI 21.7 ± 0.8 kg/m^2^. Length of menstrual cycle and duration of menstrual flow of subjects during the study were 31.5 ± 1.2 days and 6.7 ± 0.4 days, respectively. The aroma and control experiments took place on 28.7 ± 1.3th day and 29.5 ± 1.1th day in the late-luteal phase from the first day of menstruation, respectively. The interval between the two trials was 2.4 ± 0.3 days.

To confirm regular ovulatory menstrual cycles among subjects, we measured their oral temperatures and urinary ovarian hormone concentrations in the late-luteal phase (27.9 ± 1.0th day) and again in the follicular phase (7.5 ± 0.5th day), after menstruation. The basal body temperature in the late-luteal phase significantly increased from that of the follicular phase (36.54 ± 0.10 vs. 36.24 ± 0.06°C, *p* =0.012). We also found significant late-luteal increase in urinary ovarian hormones compared to the follicular phase (E1: 19.2 ± 2.7 vs. 9.2 ± 2.0 ng/ml Cr, *p* < 0.001; PdG: 1.67 ± 0.27 vs. 0.28 ± 0.05 μg/ml Cr, *p* < 0.001).

### Autonomic nervous system activity after aroma inhalation

**Figure 1:**
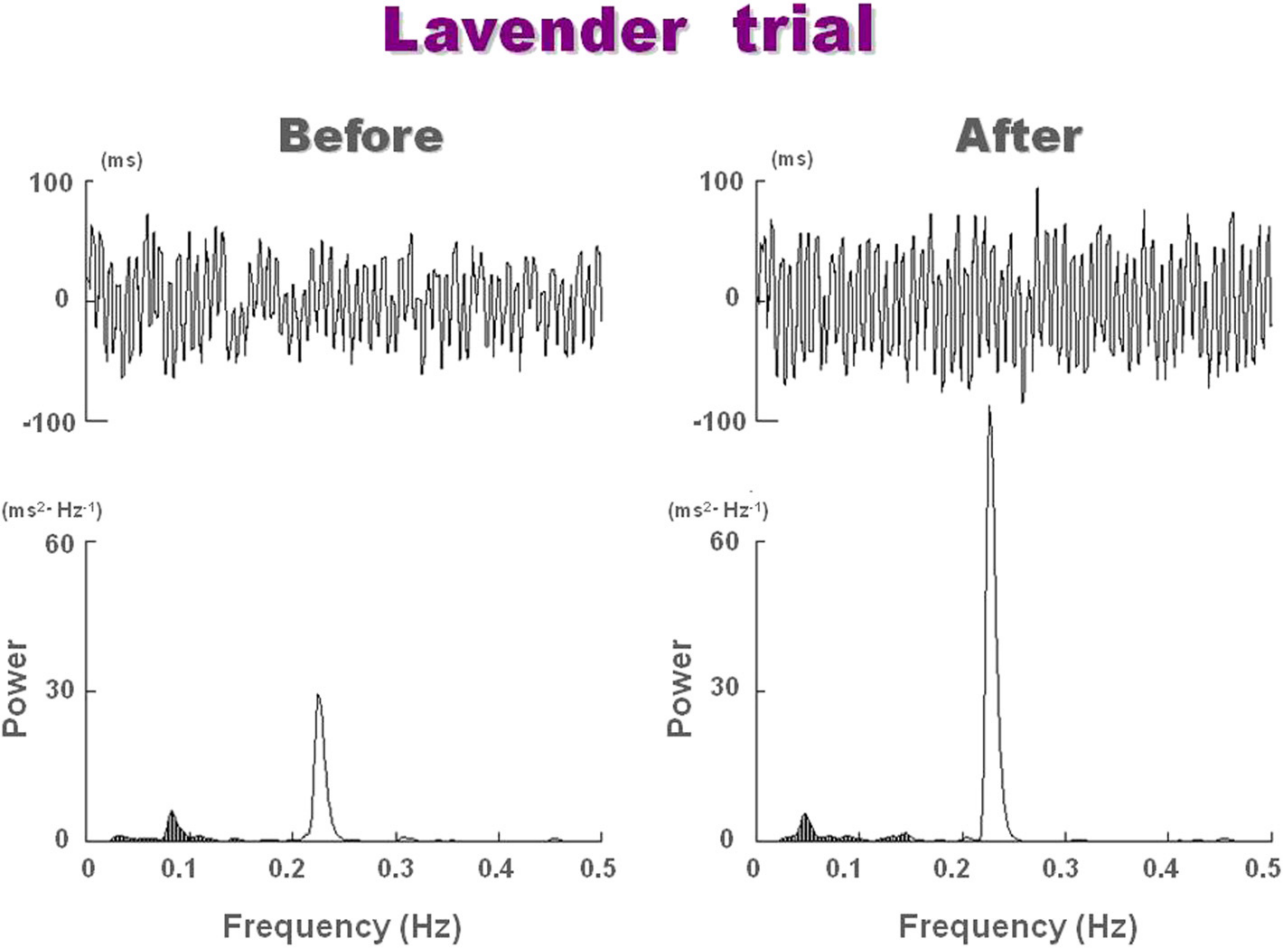
ECG R-R interval changes and corresponding power spectra before and after inhalation of lavender aroma by a 22-year-old subject.

Figure 1 represents a case of ECG R-R interval changes and the corresponding power spectra before and after inhalation of lavender aroma by a 22-year-old subject. The HF power representing the parasympathetic nervous system activity markedly increased after the inhalation of lavender.

**Figure 2:**
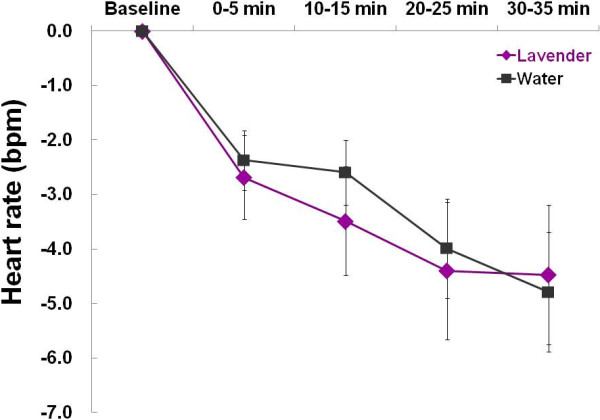
Changes of heart rates before (baseline) and after (0–35 min) inhalation of lavender aroma and water.

The baseline heart rate values before inhalation of aromas did not significantly differ between the lavender and water trials (70.6 ± 2.5 vs. 69.5 ± 2.6 bpm, *p* = 0.51). As shown in Figure 2, heart rate significantly decreased after the aroma stimulations (time effect: *F* = 12.6, *p* < 0.001), but the difference between the two trials did not reach statistical significance.

**Figure 3:**
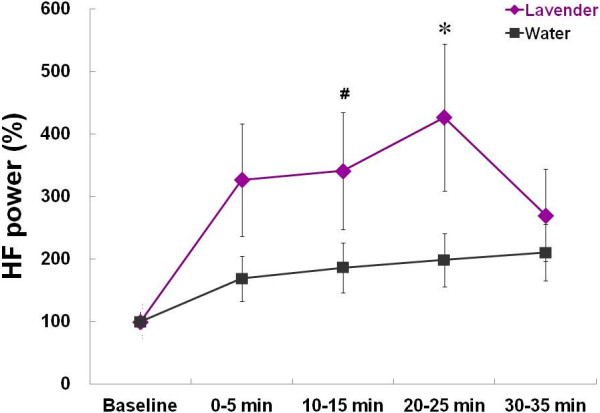
**Rate of increase of high frequency (HF) power before (baseline) and after (0–35 min) inhalation of lavender aroma and water.** A significant difference was apparent between lavender and control (water) trials (* *p* = 0.023 and ^#^*p* = 0.051).

Figure 3 graphs the time course of the changes of HF power for 35 minutes after the aroma stimulations. We observed a significant increase in the HF power after the inhalation of lavender aroma in comparison with water

 (aroma effect: *F* = 4.50, *p* = 0.050; time effect: *F* = 5.59, *p* = 0.017; aroma x time effect: *F* = 3.17, *p* = 0.047). The rate of increase of HF power was greater at 10–15 min (*p* = 0.051) and 20–25 min (*p* = 0.023) in the lavender trial compared to the control trial with water. We also calculated the ratio of LF power to HF power indicating sympathovagal balance. As suggested by Chien et al. [12], however, no statistically significant difference was detected in the relative values between the two trials.

### Premenstrual emotional symptoms after aroma inhalation

**Figure 4:**
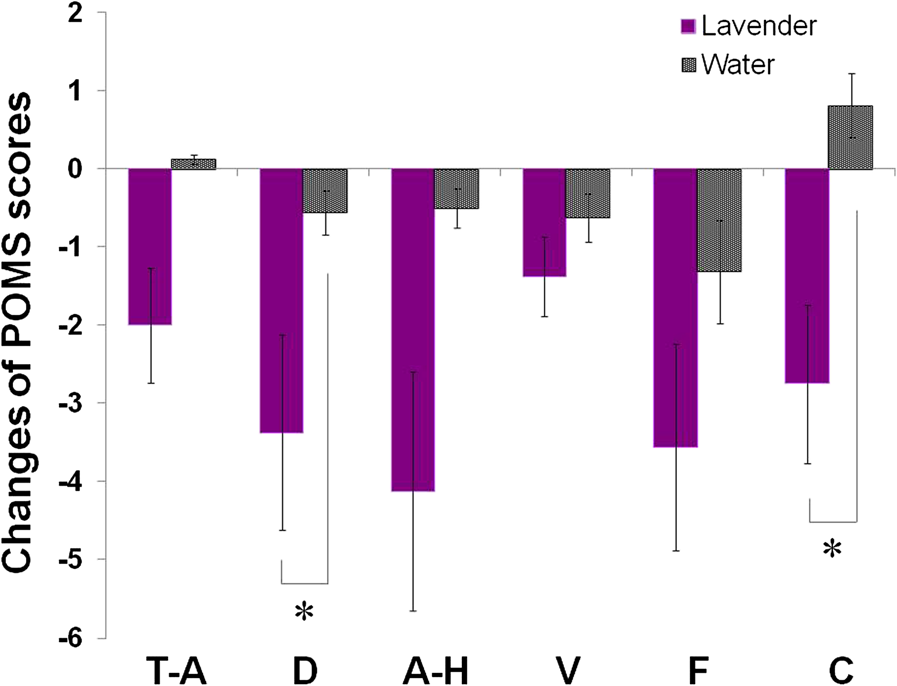
**Subscore changes in Profile of Mood States (POMS) test performed before and 35 min after inhalation of lavender aroma and water.** [Subscores: tension-anxiety (T-A), depression-dejection (D), anger-hostility (A-H), vigor (V), fatigue (F), and confusion (C).] A significant difference was apparent between lavender and control (water) trials (* *p* < 0.05).

We found no significant difference in the baseline values of six subscales of POMS test between lavender and

 control trials. The statistical results (lavender vs. control trials) are as follows: tension–anxiety 46.4 ± 2.5 vs. 44.2 ± 2.5, *p* = 0.30; depression–dejection 48.4 ± 1.9 vs. 46.4 ± 1.9, *p* = 0.19; anger–hostility 52.4 ± 3.3 vs. 47.7 ± 2.6, *p* = 0.12; vigor 44.4 ± 2.0 vs. 44.8 ± 2.2, *p* = 0.84; fatigue 47.6 ± 2.3 vs. 46.4 ± 2.7, *p* = 0.66; confusion 48.8 ± 2.1 vs. 46.7 ± 2.1, *p* = 0.18. As Figure 4 shows, however, the subscores of depression–dejection (*p* = 0.045) and confusion (*p* = 0.049) significantly decreased after the inhalation of lavender as compared to those of the control trial with water (Figure 4). Other negative symptoms—tension–anxiety, anger–hostility, and fatigue—decreased more

 in the lavender trial, but the changes of the subscores did not statistically differ between the lavender and control trials.

## Discussion

The present study investigated the effects of aromatherapy with a short-term inhalation of lavender fragrance on late-luteal autonomic nervous system activity and moderate symptom change or improvement from premenstrual emotional symptoms. Based on a literature search using the PubMed database, as of May 11, 2013, we found 756 articles on aromatherapy. However, only one article by Fukui et al. [34] addresses aromatherapy and PMS—a 20 min olfactory stimulation of saffron (*Crocus sativus*) significantly decreased salivary cortisol and increased 17-β estradiol, accompanying a significant decrease of anxiety scores in the late-luteal phase. To the best of our knowledge, the present study marks the first one to provide novel information on the psychophysiological effects of lavender on alleviating premenstrual emotional symptoms by using HRV power spectral analysis. The main findings reveal that HF power, reflecting parasympathetic nervous system activity, significantly increased directly after a 10 min inhalation of the lavender scent. This incremental effect continued at least for 25 min. In addition, the POMS test revealed that inhalation of lavender significantly decreased emotional symptoms including depression–dejection and confusion, common premenstrual symptoms, in the late-luteal phase, as long as 35 min after the aroma stimulation.

Recent research on alternative therapy has investigated both the subjective and neurophysiological effects of various plant fragrances [20]. The present study used a lavender essential oil because it has a widely applied, purportedly sedating or relaxing scent, and research has repeatedly ascribed health benefits to its aroma [6-12]. The question persists, however, how and why does lavender fragrance produce psychodynamic and physiological effects? To what extent does the autonomic nervous system, which plays a crucial role in the integrity of the mind–body connection as the functional driver of general health and wellness, contribute to efficacious outcomes produced by the fragrance? We have reviewed the literature evaluating any association of lavender and autonomic nervous system activity using HRV measurements. Saeki [35] investigated the effects of a foot-bath with and without lavender essential oil on HRV power spectra among healthy young women and demonstrated a significant increase of HF power during both types of foot-baths. The foot-bath with lavender oil, however, showed significant changes within sympathovagal balance associated with relaxation at 5 min after the completion of the foot-bath. According to Kuroda and colleagues [28], HF power increased more significantly at 21 min after a 6 min olfactory stimulation of (R)-(-)-linalool, one of the major scent components of lavender, compared to the control trials with water or (S)-(+)-linalool, the optical isomer of (R)-(-)-linalool. The study also found that after short-term inhalation, the subscores of the POMS test—tension–anxiety, depression–dejection, and anger–hostility—decreased to a greater extent than in the control trials. Chien et al. [12] investigated the effects of 12 weeks of lavender aromatherapy in midlife women (45–55 years of age) with insomnia. This study observed a significant increase in HF power after a 20 min lavender inhalation in the 4th and 12th weeks of aromatherapy and also found an apparent improvement in sleep quality after the intervention. Despite the differences in experimental designs and conditions, these earlier investigations support our findings indicating that short-term lavender inhalation modulates HRV with the predominance of parasympathetic nervous system activity while inducing soothing effects.

While we mentioned the paucity of information on the effects of lavender inhalation and PMS, one study by Shimizu [29] published in a Japanese medical journal reported on the negative subscales of the POMS test—tension–anxiety, depression–dejection, anger–hostility and confusion—significantly decreasing and a positive subscore, vigor, increasing in the luteal phase after therapeutic intervention with a 15 min inhalation of linalyl acetate, another major component of lavender, twice a day (morning and evening) daily for one menstrual cycle. This study did not measure any physiological data including HRV measurements. Considering three previous investigations mentioned above [12,28,35] and the present research together with the findings by Shimizu [29], however, lavender could serve as a potential anti-PMS fragrance, especially for alleviating negative emotional stress appearing premenstrually. Although the detailed mechanism of the efficacy of lavender on premenstrual symptoms remains unknown, the significant increase in HF power after the inhalation of lavender found in the present study further suggests that lavender interacts with parasympathetic nervous system activity to modulate the cluster of premenstrual psychoemotional symptoms.

From a pharmacological point of view, Herz [20] showed that lavender acts postsynaptically and suggested that lavender modulates the activity of cyclic adenosine monophosphate (cAMP). A reduction in cAMP activity is associated with sedation. Linalool, a principal component of lavender, has also been found to inhibit glutamate binding, which may have sedative effects. To further explore the psychoneurophysiological mechanism of aromatherapy with lavender, neuroimaging techniques provide new insights into the role of the brain in correlation with autonomic modulation. Olfaction is mediated by chemoreceptors of olfactory cells located in the nasal mucosae and olfactory neurons in the olfactory bulb. Olfactory information is further projected to the primary olfactory regions in the brain and most of these brain regions are strongly connected to or are part of the limbic system, the center of autonomic function and emotion [6]. By applying a combination of HRV power spectral analysis and positron emission tomography (PET) examination, Duan and colleagues [36] found a significant increase in HF power, indicating parasympathetic nervous system acidity after a lavender aroma treatment, as we demonstrated in the present study. Simultaneous PET measurements, detecting the specific regional metabolic activations and reductions after the treatment, suggested that the lavender fragrance induced not only physical relaxation but also improved mental function.

The present study used two kinds of aroma, lavender and water as a control. To avoid placebo effects, we did not inform subjects of which fragrance we would use for the experiment or that one of the two trials could be a control trial, during the entire study period. We cannot, however, completely deny the possibility that the participants would have noticed the difference when they inhaled the aroma of water. A study using lavender together with other plant fragrances possessing different psychological and pharmacological effects could extend the present research by scrutinizing the effects of aromatherapy on premenstrual symptoms while protecting against a placebo effect.

While this study produced intriguing findings—augmentation of parasympathetic nerve activity accompanying sedative effects on negative premenstrual emotional status by lavender stimulation—it employed a relatively small sample size. In addition, the intensity of premenstrual symptoms among subjects ranged from mild to moderate and represents the premenstrual molimina experienced by a greater percentage of reproductive age women, compared to PMS or PMDD [1-3]. Our previous research has revealed that the menstrual cyclic patterns of autonomic nervous system activity significantly differ among women with premenstrual molimina, PMS and PMDD [21-24]. We thus need to conduct future studies with a larger sampling of women with different degrees of symptoms—from premenstrual molimina to PMDD together with premenstrual exacerbation [26]—to further explore the therapeutic efficacy of plant fragrances including lavender on premenstrual symptomatology. Premenstrual discomfort remains enigmatic, but researchers continue to unveil its etiology, which includes estrogen excess, progesterone deficiency, decreases in serotonergic tone, alteration of central GABA function, and opioids withdrawal [1-3,26]. These theoretical models lead us to speculate that the sophisticated independent menstrual-cyclic fluctuation of ovarian hormones, estrogen and progesterone, could ingeniously interact with the central nervous, autonomic nervous, endocrine and immune systems. Taken together, interdisciplinary research is also required to scrutinize the psychological, neurophysiological, and pharmacological functions of lavender essential oils for alleviating the undefined symptom complex appearing so commonly premenstrually.

In conclusion, the present study, using HRV power spectral analysis, investigated the efficacy of lavender aromatherapy as a potential therapeutic modality of premenstrual symptoms from the perspective of autonomic nervous system activity. This study indicates that short-term inhalation of lavender could alleviate premenstrual emotional symptoms and could, at least in part, contribute to the improvement of parasympathetic nervous system activity. The present study further implies that HRV can be used to evaluate the efficacy of aromatherapy (using various kinds of fragrances) in relieving premenstrual symptoms, and ultimately, promote the mind and body health of women.

## Competing interests

The authors declare that they have no competing interest.

## Authors’ contributions

TM conceptualized and designed the study, collected and analyzed the data, performed the statistical analysis, interpreted the results, and drafted the manuscript. HA participated in the design and coordination of the present study and provided clinical evidence of premenstrual symptoms from his gynecological research as well as practical suggestions to interpret the results. TH contributed to analyzing data, helped to interpret the results with productive and valuable comments, and provided neuroendocrinological information to develop the present research. All authors read and approved the final manuscript.
